# Increased CEACAM1 expression on peripheral blood neutrophils in patients with rheumatoid arthritis

**DOI:** 10.3389/fimmu.2022.978435

**Published:** 2022-12-14

**Authors:** Haruki Matsumoto, Yuya Fujita, Michio Onizawa, Kenji Saito, Yuya Sumichika, Shuhei Yoshida, Jumpei Temmoku, Naoki Matsuoka, Makiko Yashiro-Furuya, Tomoyuki Asano, Shuzo Sato, Eiji Suzuki, Takeshi Machida, Hiroshi Watanabe, Kiyoshi Migita

**Affiliations:** ^1^ Department of Rheumatology, Fukushima Medical University School of Medicine, Fukushima, Fukushima, Japan; ^2^ Department of Gastroenterology, Fukushima Medical University School of Medicine, Fukushima, Fukushima, Japan; ^3^ Department of Immunology, Fukushima Medical University School of Medicine, Fukushima, Fukushima, Japan

**Keywords:** rheumatoid arthritis, CEACAM1, TIM–3, neutrophils, monocytes

## Abstract

Altered expression of adhesion molecules in immune cells has been demonstrated in rheumatoid arthritis (RA). Carcinoembryonic–antigen–related cell–adhesion molecule 1 (CEACAM1) is an adhesion molecule that acts as a coinhibitory receptor in the immune system. We investigated the role of CEACAM1 in immune cell subsets of patients with RA. Peripheral blood was obtained from 37 patients with RA and 20 healthy controls (HC). The expression of CEACAM1 and T–cell immunoglobulin mucin domain molecule (TIM) –3 on peripheral blood mononuclear cells and neutrophils was analyzed by flow cytometry. Intracellular TIM–3 expression was analyzed using cellular lysates by Western blot analysis. Serum levels of soluble CEACAM1 (sCEACAM1) were estimated by an enzyme-linked immunosorbent assay. CEACAM1 expression was not detected in peripheral blood mononuclear cells, including in CD14(+) monocytes and CD3(+) lymphocytes isolated from patients with RA or HC. However, substantial cell–surface expression of CEACAM1 was detected in peripheral blood neutrophils, and it was significantly elevated in samples from patients with RA without remission compared to those in remission. There was no significant difference in serum levels of sCEACAM1 between patients with RA and HC. Cell-surface expression of TIM-3 was not detected in peripheral blood neutrophils from patients with RA or HC but was seen in CD14(+) monocytes. However, there was no significant difference in TIM–3 expression on monocytes between patients with RA and HC. Our data indicate that cell-surface expression of CEACAM1 on peripheral blood neutrophils are higher in patients with RA and that it is associated with rheumatoid inflammation. Further studies are needed to explore the potential role of CEACAM1 in rheumatoid inflammatory pathways.

## 1 Introduction

Rheumatoid arthritis (RA) is a chronic systemic autoimmune disorder that is characterized by inflammatory cell infiltrations into the synovial tissues, which results in the joint destruction (1). The rheumatoid synovium is also characterized by the increased expression of adhesion molecules of the infiltrating immune cells (2), and ligand binding on these adhesion molecules induces immune cell activation and inflammatory cytokine production (3). Carcinoembryonic–antigen–related cell–adhesion molecule 1 (CEACAM1), also known as cluster of differentiation (CD) 66a, is an adhesion molecule expressed by immune cells that interact with integrin or extracellular matrix proteins to modulate the immune response (4). Thus, CEACAM1 possesses diverse biological functions in cell adhesion, migration, and intercellular signaling during inflammation (5). Also, CEACAM1 is thought to be a coinhibitory molecule in the immune system (6) as it acts synergistically with TIM–3, which is a negative regulatory molecule that plays a critical role in immune tolerance (7). We have previously demonstrated that serum levels of soluble TIM–3 (sTIM–3) are elevated in patients with RA and that it is associated with proinflammatory markers and RA disease activity (8). Recently, it was demonstrated that CEACAM1 serves as a heterophilic ligand for TIM–3 in activated T cells and that this interaction is crucial for regulating autoimmunity (9). These findings suggest that CEACAM1 also plays a functional role in T cells during inflammatory or autoimmune disorders (10). CEACAM1 expression was shown to be up-regulated on the innate immune cells when cells are activated by pathogens (11). Furthermore, CEACAM1 expressing myeloid cells control angiogenesis suggesting a causal relation between CEACAM1 expression and its inhibitory effect during the inflammatory processes (12). However, very few studies have investigated the role of CEACAM1 in regulating autoimmunity in human autoimmune diseases. Hence, we evaluated CEACAM1 and TIM–3 expression in peripheral blood immune cells isolated from patients with RA by flow cytometry and also investigated the relationship between their expression and rheumatoid inflammatory parameter in patients with RA.

## 2 Methods

### 2.1 Patients

This single–center study observational study included 37 patients with RA who were diagnosed based on the 2010ACR/EULAR classification criteria (13). RA patients were enrolled between August 2021 and March 2022, and we reviewed the records of these patients with RA. All patients were treated at the Department of Rheumatology, Fukushima Medical University.

The following clinical and demographic data were retrieved from the Medical Records Unit of the Fukushima University Hospital: age, gender, simplified disease activity score (SDAI), and disease activity score–28 for RA with C-reactive protein (DAS28–CRP). For comparison with healthy controls (HC), 20 individuals, i.e., 6 males and 14 females with a median age of 44.5 years at blood test [interquartile range (IQR); 30–51.25 years], were included. This study conformed to the principles of the Declaration of Helsinki. Ethical approval for this study (No.2021–158) was provided by the Ethics Committee of Fukushima Medical University.

### 2.2 Measurement of clinical disease activity

All patients underwent clinical assessment at baseline, including 28–swollen joint and tender joint counts (28–SJC and 28–TJC, respectively), physician and global patient assessment with visual analogue scales (0–100 mm), and CRP (mg/dL). Composite disease activity indices were subsequently calculated based on SDAI and DAS28–CRP. This score was reported as a quantitative value that could be categorized as remission and non-remission (low, intermediate, and high disease activity).

### 2.3 Blood sampling and cell isolation

Peripheral blood (2 mL) from patients with RA and HC was collected into precoated EDTA tubes, diluted with 6 ml of cold Ammonium–Chloride Potassium (ACK) lysis buffer (0.15 M NH_4_Cl, 13 mM KCl, 0.1 mM Na_2_EDTA, pH 7.4) (14), and incubated for 5 min on ice. A cell pellet was obtained by centrifugation (10 min, 3,000 ×*g*, 4°C) and washed once with 6 ml cold ACK buffer. The supernatant was discarded after the last centrifugation step and the cell pellet was resuspended in cold PBS.

### 2.4 Neutrophil isolation and cytokine stimulation

HC`s neutrophils were isolated on the basis of the density gradient centrifugation method. In brief, peripheral blood was layered on a Polymorphprep™ (Axis-Shield, Oslo, Norway) cushion, and cells were isolated according to the manufacturer’s protocol. Isolated neutrophils were stimulated with IL-6 (20 ng/mL and 100 ng/mL) and TNF-α (20 ng/mL and 100 ng/mL) for 24 hr *in vitro*. Cell surface expressions of CEACAM1 on neutrophils were analyzed by flow cytometry.

### 2.5 Flow cytometry

Freshly isolated white blood cells were used for flow cytometric analysis of the surface expression of CEACAM1, TIM–3, CD3, CD14, and CD16 using antihuman CEACAM1 (PE; mouse. No. 283340, diluent 2:100; R&D Systems, Minneapolis, MN, USA), anti–TIM3 (APC; rat. No. 344823, diluent 2:100; R&D Systems), antihuman CD3 (PerCP–CY5.5; mouse. No. 344806, diluent 2:100; Biolegend, San Diego, CA, USA), antihuman CD14 (FITC; mouse. No.301804, diluent 2:100; Biolegend), and antihuman CD16 antibodies (PerCP; mouse. No. 302029, diluent 2:100; Biolegend), respectively. Dead cells were excluded using LIVE/DEAD™ Fixable Dead Cell Stain Kits. Anti–CEACAM1–specific mouse monoclonal antibody (clone number 283340) was shown to be non-cross-reactive with other CEACAM families as described previously (15). Flow cytometry was performed on a FACS–Canto II (BD Biosciences, Franklin Lakes, NJ, USA) and data were analyzed using FlowJo software (BD Biosciences). Flow cytometry gating strategies were shown in **Supplemental Figures 1**, **2**.

### 2.6 ELISA methods

Serum concentration of CEACAM1 was measured using enzyme–linked immunosorbent assay (ELISA) kits (Thermo Fisher Scientific, Waltham, MA, USA; Catalog No. EHCEACAM1) according to the manufacturer’s instructions. Similarly, serum TIM–3 was measured using an ELISA Kit (R&D Systems; Catalog No. DTIM30). The CEACAM1 ELISA kit uses the solid-phase sandwich enzyme immunoassay technique, and the TIM–3 ELISA kit uses the quantitative sandwich enzyme immunoassay technique. The CEACAM1 ELISA kit involves specific detection of captured target proteins using biotinylated detection antibody and the interaction was then analyzed using streptavidin conjugated with horseradish peroxidase according to the manufacturer’s protocol. Monoclonal antibodies specific for CEACAM1 and TIM–3 have been pre-coated onto a microplate. Standards and samples are pipetted into the wells and any CEACAM1 and TIM–3 present are bound by the immobilized antibody. After washing away any unbound substances, enzyme–linked polyclonal antibodies specific for CEACAM1 and TIM–3 are added to the wells. Following a wash to remove any unbound antibody–enzyme reagent, a substrate solution is added to the wells and color develops in proportion to the amounts of CEACAM1 and TIM–3 bound in the initial step. The color development is stopped and the intensity of the color is measured. Determine the optical density of each well within 30 minutes using a microplate reader set to 450 nm.

### 2.7 Western blotting for intracellular TIM–3 expression

Freshly isolated neutrophils were washed in ice–cold PBS and lysed on ice for 20 min with RIPA buffer (Sigma–Aldrich, La Jolla, CA, USA) supplemented with protease inhibitor cocktail. Cell lysates were centrifuged at 10,000×*g* for 10 min at 4°C and the supernatant was collected. Equal amounts of total protein (30 μg) were subjected to 12% SDS–PAGE under reducing condition and electrotransferred onto polyvinylidene fluoride membranes that were then blocked with 5% bovine serum albumin for 1 h at room temperature. The membrane was incubated with primary antibodies against human TIM–3, followed by incubation with secondary antibodies at room temperature and visualization using the ECL reagent (Amersham, Little Chalfont, UK). Immunoblots were imaged using the LAS–3000 Imaging System (Fuji Film, Tokyo, Japan). β–actin was also detected to normalize the loading amount of total cell lysate between samples.

### 2.8 Statistical analyses

Nonnormally distributed data are presented throughout the manuscript as median with 25–75^th^ centiles and were compared by Mann–Whitney *U* test. Pearson’s Chi-square tests were performed for dichotomic variables. Correlation between continuous variables was analyzed using the Spearman rank correlation test. All data entry and statistical analyses were performed using SPSS Statistics version 22.0 (IBM, Armonk, NY, USA). All statistical analyses were 2–tailed, and statistical significance was defined as *p* < 0.05. Mean fluorescence intensity (MFI) of any given marker within the defined immune cells subpopulation were recorded.

## 3 Results

### 3.1 Characteristics of patients with RA

The demographic, clinical, and laboratory characteristics of the 37 patients with RA are summarized in **Table 1**. The median age (IQR) at blood test for this group was 69 years (58–77 years). Among 37 patients with RA, 34 (91.9%) were female. The median values (IQR) for SDAI and DAS28–CRP were 4.12 (2.03–13.13) and 1.93 (1.28–2.97) respectively. Patients with RA were classified into stages I to IV according to Steinbrocker radiographic staging (16), and there were 7 (19%) in stage I, 9 (24%) in stage II, 7 (19%) in stage III, and 12 (32%) in stage IV. The staging was not possible in some patients (n=2) due to a lack of radiological information.

**Table 1:** Baseline characteristics of 37 Japanese patients with RA.

Characteristics	Value
Age at diagnosis (years), median (IQR)	54 (39–60)
Age at time of blood test (years), median (IQR)	69 (58–77)
Female, n (%)	34 (91.9)
Duration of RA (year), median (IQR)	10 (5–21)
CRP (mg/dL), median (IQR)	0.2 (0.01–1.11)
RF (IU/mL), median (IQR)	83 (28–204)
Anti CCP–Ab (U/mL), median (IQR)	72.5 (11.7–500)
SDAI, median (IQR)	4.12 (2.03–13.13)
DAS28–CRP, median (IQR)	1.93 (1.28–2.97)
Biologics, n (%)	13 (35)
Anti–TNF–α Ab, n (%)	1 (2)
Anti–IL–6 receptor Ab, n (%)	10 (27)
Selective T Cell Co–stimulation Modulator, n (%)	2 (5)
Janus Kinase inhibitor, n (%)	4 (11)
Steinbrocker stage	I:7, II:9, III:7, IV:12

Ab, antibody; CCP, cyclic citrullinated peptide; CRP, C reactive protein; DAS28, Disease Activity Score; IL, interleukin; IQR, interquartile range; RF, rheumatoid factor; SDAI, simplified disease activity index; TNF, tumor necrosis factor.

### 3.2 Expression of CEACAM1 on blood cells

**Figure 1:**
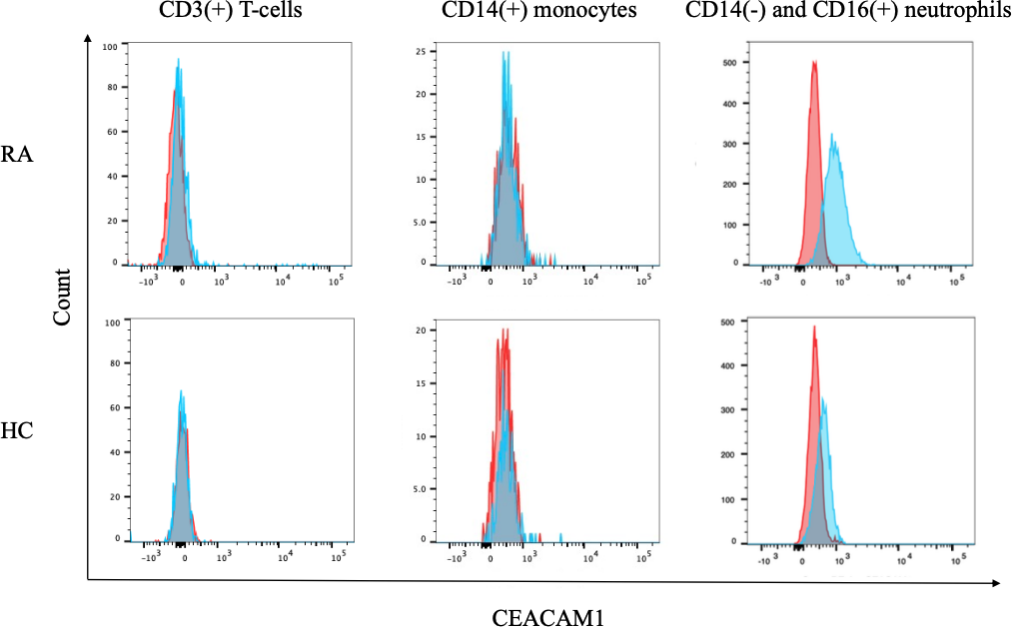
The expression of CEACAM1 on peripheral blood immune cells. Flow cytometry overlay histograms for CEACAM1 demonstrating typical cell profiles from subdivided peripheral blood immune cells from RA patients and HC. CEACAM1 expression was detected using anti–CEACAM1 antibody (blue shaded histogram) or isotype control antibody (red shaded histograms). In contrast to lymphocytes or monocytes, peripheral blood neutrophils expressed CEACAM1 on their surface. RA, rheumatoid arthritis; HC, healthy controls.

Anticoagulated whole blood from patients with RA and HC were used to evaluate the expression of CEACAM1 on peripheral immune cells. As shown in representative images, minimal CEACAM1 expression was seen on the cell surface of CD3(+) lymphocytes and CD14(+) monocytes (**Figure 1**). In contrast, CEACAM1 was expressed on the surface of CD16(+) CD14 (–) neutrophil populations (**Figure 1**). These results indicate that cell–surface expression of CEACAM1 is seen in the neutrophils in human peripheral blood.

### 3.3 Evaluation of CEACAM1 expression on neutrophils

**Figure 2:**
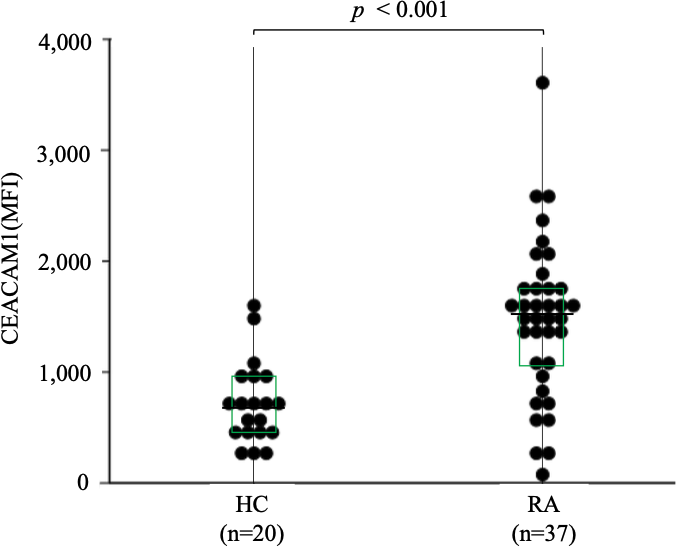
The expression of CEACAM1 on peripheral blood neutrophils isolated from HC (n=20) and RA patients (n=37). The MFI of CEACAM1 in RA patients was significantly higher compared to those in HC. Statistical significance was determined by the Mann–Whitney *U* test. Horizontal bars and squares indicate median and IQR, respectively. HC, healthy controls; RA, rheumatoid arthritis; MFI, mean fluorescence intensity; IQR, interquartile range.

**Figure 3:**
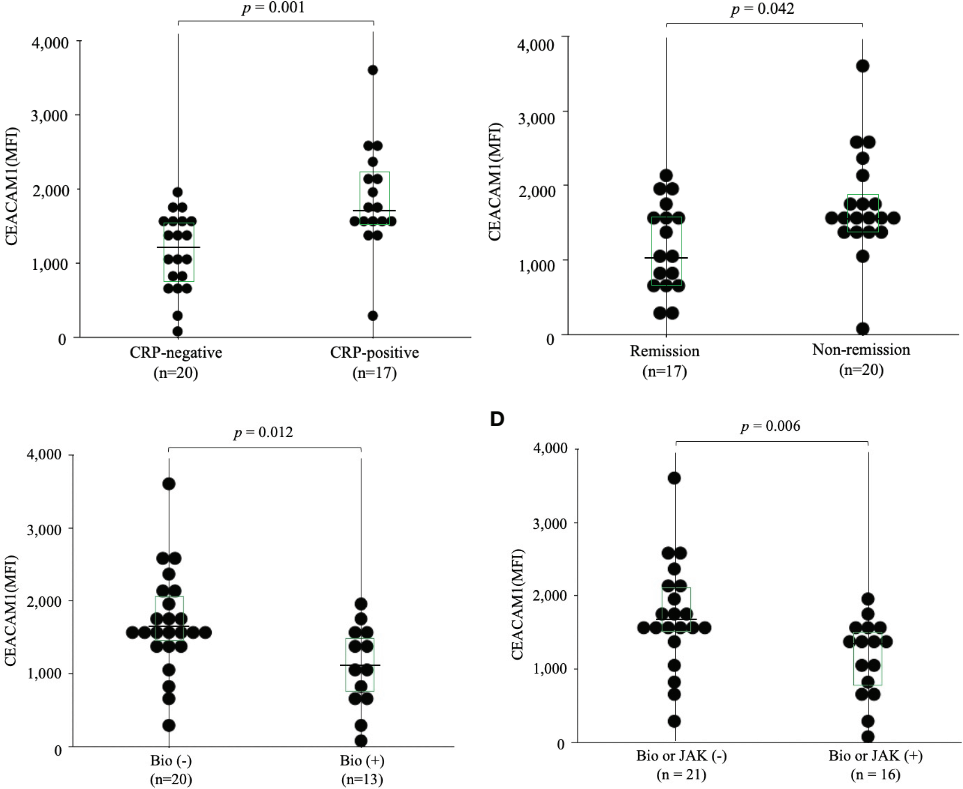
The expression of CEACAM1 on peripheral blood neutrophils in RA patients. **(A)** The expression of CEACAM1 on peripheral blood neutrophils isolated from RA patients with (n=17) or without (n=20) elevated serum inflammatory marker (CRP >0.30 mg/dL). The MFI of CEACAM1 positive neutrophils in RA patients with elevated serum inflammatory marker was significantly higher compared to RA patients without them. **(B)** The expression of CEACAM1 on peripheral blood neutrophils in RA patients with or without remission according to SDAI. The MFI of CEACAM1 in the non–remission group was significantly higher compared to the remission group. **(C, D)** The MFI of CEACAM1 was significantly higher in the RA patients who used Bio or JAK inhibitors than in the non-used patients. Statistical significance was determined by the Mann–Whitney *U* test. Horizontal bars and squares indicate the median and IQR, respectively. RA, rheumatoid arthritis; CRP, C–reactive protein; MFI, mean fluorescence intensity; Bio, Biologics; JAK, Janus kinase; IQR, interquartile range.

**Figure 4:**
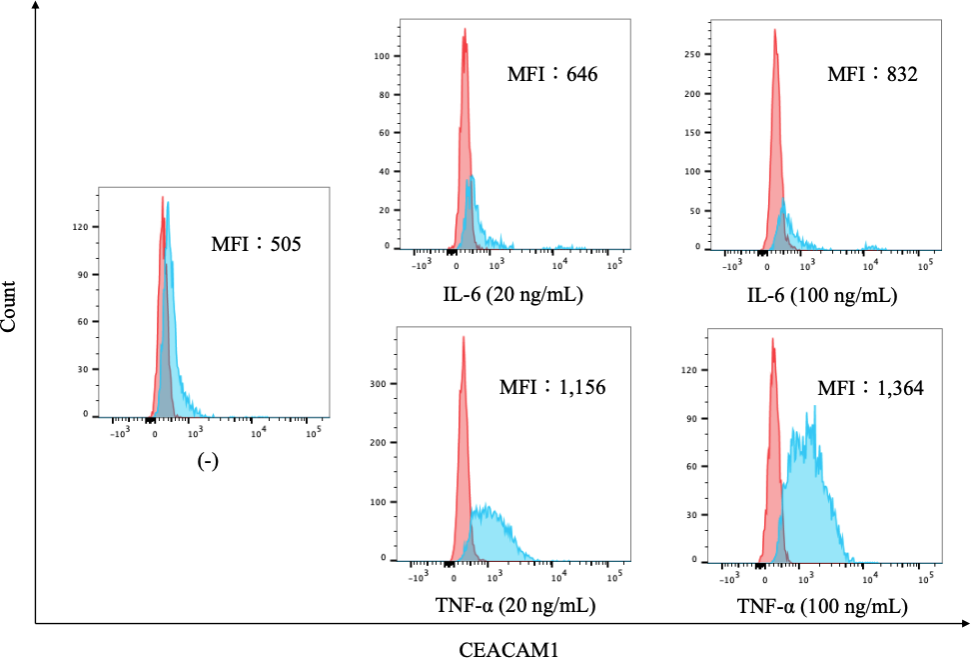
The expression of CEACAM1 on cytokine-stimulated neutrophils. Flow cytometry overlay histograms for CEACAM1 demonstrating typical cell profiles from subdivided peripheral blood immune cells from HC. CEACAM1 expression was detected using anti–CEACAM1 antibody (blue shaded histogram) or isotype control antibody (red shaded histograms). Neutrophils were stimulated with cytokines associated with inflammatory pathogenesis of RA [IL-6 (20 ng/mL and 100 ng/mL) and TNF-α (20 ng/mL and 100 ng/mL)]. The MFI of CEACAM1 differed depending on the cytokine and its concentrations. HC, healthy controls; IL-6, interleukin-6; TNF-α: tumor necrosis factor; MFI, mean fluorescence intensity.

To further evaluate the expression of CEACAM1 on neutrophils, we compared the expression levels of CEACAM1 in peripheral blood neutrophils from 20 HC and 37 patients with RA. As shown in **Figure 2**, the MFI of CEACAM1 (median 1,534, IQR 1,055–1,748) was significantly higher in patients with RA compared to HC (median 696, IQR 438.5–936) (*p* < 0.001). Additionally, the MFI of CEACAM1 were significantly higher in the CRP–positive RA group (median 1603, IQR 1,434–1,920) than in the CRP–negative RA group (median 1,534, IQR 1,055–1748) (*p* = 0.001) (**Figure 3A**). Furthermore, we investigated the differences in CEACAM1 expression in neutrophils between RA patients with and without remission state. The MFI of CEACAM1 was significantly higher in the non-remission group than in the remission group for SDAI (**Figure 3B**). The clinical background between the remission group and non-remission group were shown in **Supplemental Table 1**. However, there was no significant difference between the remission group and the non-remission group for DAS28–CRP (**Supplemental Figure 3**). The MFI of CEACAM1 was not correlated with SDAI or DAS28–CRP (**Supplemental Figure 4**), even though the association with disease activity was not clear. Furthermore, the MFI of CEACAM1 was significantly lower in the RA patients who used biologics or Janus kinase (JAK) inhibitors compared to the non-used patients (**Figures 3C, D**). Elevated levels of inflammatory cytokines such as TNF-α and IL-6 in RA patients are thought to play a crucial role in the development and progression of RA. To analyze whether these proinflammatory cytokines modulate the CEACAM1 expressions of neutrophils, peripheral blood neutrophils were simulated with TNF-α or IL-6 for 24 hr *in vitro*. Cell surface expressions of CEACAM1 on neutrophils were analyzed by FCM. The expression of CEACAM1 levels was marginally elevated in IL-6-stimulated neutrophils. However, levels of CEACAM1 expression were significantly up-regulated in TNF-α-stimulated neutrophils (**Figure 4**). As shown in **Supplemental Figure 5**, after removing the dead cells, the expression of CEACAM1 levels was elevated in live cells.

### 3.4 Evaluation of sCEACAM1 levels

**Figure 5:**
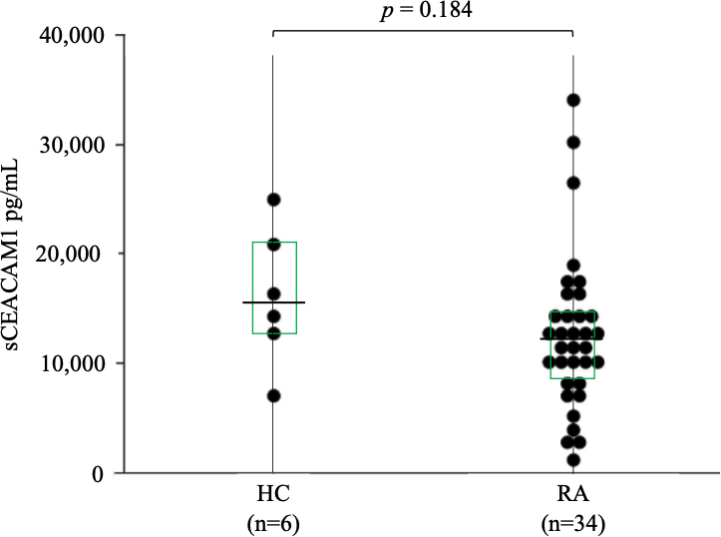
Serum levels of sCEACAM1 in RA. The comparison of serum levels of sCEACAM1 between HC (n=6) and RA patients (n=34). There were no significant differences between HC and RA patients. Statistical significance was determined by the Mann–Whitney *U* test. Horizontal bars and squares indicate median and IQR, respectively. sCEACAM1, soluble CEACAM1; HC, healthy controls; RA, rheumatoid arthritis; IQR, interquartile range.

Levels of sCEACAM1 were measured using serum samples from patients with RA and HC, and as shown in **Figure 5**, there were no significant differences between the two groups [12,060 (8,622–14,556) pg/mL versus 15486 (13,014–19,865) pg/mL, *p* = 0.184]. CEACAM1 is known to interact with TIM–3 during immune regulation; hence, its correlation with serum TIM–3 levels were also investigated, but there was no significant correlation (the data was not shown).

### 3.5 Expression of TIM–3 on peripheral blood immune cells

**Figure 6:**
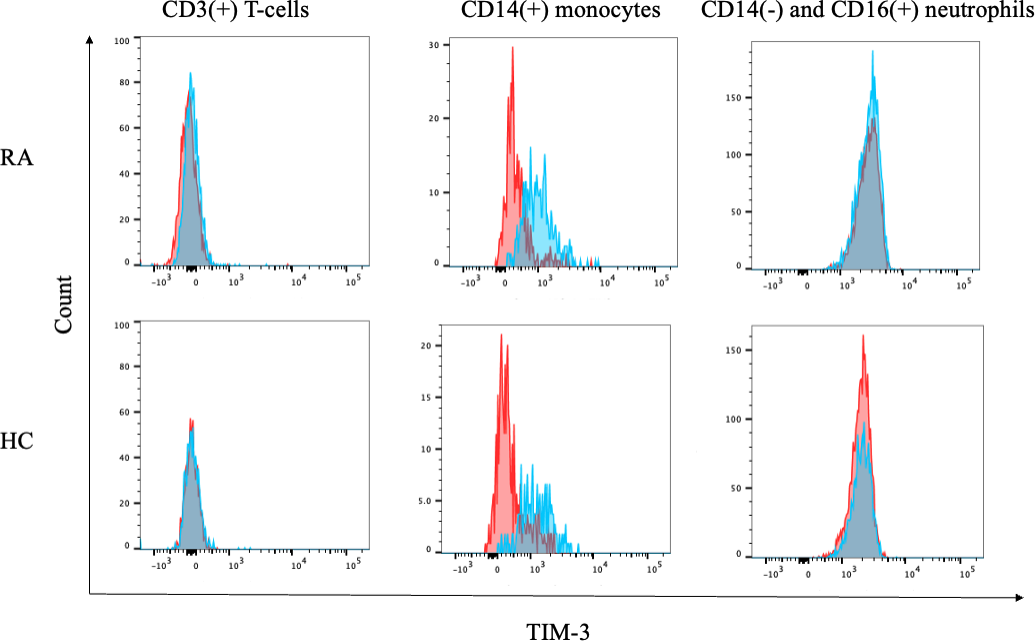
Expression of TIM–3 on blood cells. Flow cytometry overlay histograms for TIM–3 demonstrating typical cell profiles from subdivided peripheral blood cells from RA patients and HC. TIM–3 expression was detected using anti-TIM–3 antibody (blue shaded histogram) or isotype control antibody (red shaded histograms). In contrast to lymphocytes or neutrophils, peripheral blood monocytes expressed CEACAM1 on their surface. RA, rheumatoid arthritis.

To assess TIM–3 expression in RA, we subjected fresh peripheral blood samples from HC and patients with RA to flow cytometry and found that TIM–3 is barely expressed on peripheral blood CD3(+) T cells or CD14(−) and CD16(+) neutrophils, both in HC and patients with RA (**Figure 6**). To analyze the expression of TIM–3 on monocytes, CD14(+) monocytes in HC and patients with RA were gated, and the surface expression of TIM-3 was analyzed (**Supplemental Figure 1**). While we observed TIM–3 expression on peripheral blood CD14(+) monocytes, there was no significant difference in TIM–3 expression levels between HC and patients with RA (**Figure 6**).

### 3.6 Intracellular expression of TIM–3 in neutrophils

**Figure 7:**
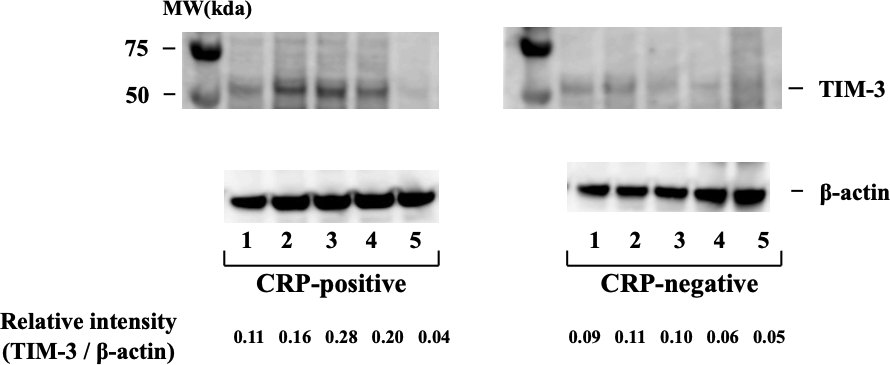
Intracellular expression of TIM–3 in neutrophils isolated from RA patients with or without inflammatory sign (CRP >0.30 mg/dL). Intracellular expression of TIM–3 was determined by Western blotting using neutrophils cellular lysate in RA patients with or without elevated serum CRP levels. TIM–3 expression was normalized β–actin bands and the ratio of TIM–3 to β–actin was calculated. They indicate that the intracellular expression of TIM–3 was similar between active and inactive patients with RA. RA, rheumatoid arthritis; CRP, C-reactive protein.

As flow cytometry did not detect cell-surface expression of TIM–3 on peripheral blood neutrophils, we analyzed its intracellular expression using neutrophil cellular lysates by Western blot. Whole neutrophil lysates displayed bands characteristic of TIM–3 (**Figure 7**), and in agreement with the previous reports on the molecular mass of TIM–3, the fully glycosylated form of the protein (64 kDa) was found in neutrophils (17). The results indicate that the intracellular expression of TIM–3 was similar between active and inactive patients with RA.

## 4 Discussion

The rheumatoid synovium displays the increased expression of adhesion molecules on the infiltrating immune cells (18). Importantly, binding of these adhesion molecules to the cells in the synovial microenvironment can induce immune cell activation (19). CEACAM1, also known as CD66a, is a member of the carcinoembryonic antigen family and is widely expressed in different cell types (4). It is involved in intercellular adhesion and in the regulation of cellular growth and differentiation, and when expressed on cell membranes, it interacts with integrin or extracellular matrix proteins to modulate immune response (5). However, as little is known about the role of CEACAM1 in rheumatoid inflammatory cells, we investigated its expression in peripheral blood immune cells isolated from RA patients with systemic inflammation. We demonstrate that CEACAM1 is unlikely expressed on monocytes or lymphocytes, while it is exclusively expressed on peripheral blood neutrophils in RA patients. Furthermore, cell-surface expression of CEACAM1 was significantly higher in neutrophils isolated from patients with RA and systemic inflammation compared to those without systemic inflammation as well as HC.

Many studies have evaluated the expression of various activation–dependent surface markers in neutrophils isolated from the synovial fluid of patients with inflammatory arthritis (20) and they report greater expression of members from the CD66 family in neutrophils from the synovial fluid compared to peripheral blood neutrophils (21). The enhanced expression of CEACAM1 on neutrophils in patients with RA suggests that CEACAM1 expression can be modulated by the rheumatoid inflammatory process. Nevertheless, this should be further validated by investigating the relationship between CEACAM1 expression and interacting molecules or immune cells seen during the rheumatoid inflammatory process.

Neutrophils are regarded as the first line of defense pathogens (22), and while much is known about their migration to inflamed tissue, knowledge of their regulation during the inflammatory process is relatively scant (23). In rheumatoid synovium, activated neutrophils secrete inflammatory cytokines, which are implicated in RA pathogenesis (24). Also, circulating neutrophils isolated from RA patients were shown to exhibit several features indicative of partial activation (25). Taken together, our data suggest that inflammatory cytokines can be implicated in the increased CEACAM1 expressions on circulating neutrophils in RA. CEACAM1 is one of the adhesion molecules on neutrophils that plays an important role in the cell’s biological functions, including adhesion or phagocytosis (26). Initially, there is little CEACAM1 expression on resting human neutrophils but their activation causes rapid translocation of CEACAM1 to the cell surface (27). Although CEACAM1 is an activation marker for neutrophils, its role in neutrophil-dependent inflammation is not completely elucidated. In our data, RA patients with inflammation exhibit an increased expression of CEACAM1 on their peripheral blood neutrophils. In contrast, CEACAM1 expression is barely seen in peripheral blood neutrophils of HC, and these observations support the hypothesis that inflammatory stimuli upregulate CEACAM1 expression on neutrophils. The levels of CEACAM1 expression were significantly up-regulated in TNF-α-stimulated neutrophils (**Figure 4**). Indeed, the MFI of CEACAM1 was significantly lower in the RA patients treated with cytokine-targeting treatments compared to those without these treatments. In rheumatoid synovium, activated neutrophils secrete many cytokines and are implicated in RA pathogenesis (24). Also, circulating neutrophils isolated from RA patients were shown to exhibit several features indicative of partial activation (25). Taken together, our data suggest that inflammatory cytokines can be implicated in the increased CEACAM1 expressions on circulating neutrophils in RA. To the best of our knowledge, this is the first report of describing the role of CEACAM1 in rheumatoid inflammatory processes.

Although CEACAM1 is designated as an activation marker in immune cells, it also acts as a co-inhibitory receptor in the immune system (11). CEACAM1 is an ITIM type 1 membrane protein and its main functional role in lymphocytes is to generate inhibitory signals (28); however, the inhibitory effects of CEACAM1 have not been completely elucidated. CEACAM1 can bind to TIM–3 intracellularly, which appears to be important for the function of TIM–3 (29). *In vitro*, CEACAM1 is co–expressed with TIM–3 in activated T cells and is thought to contribute to the establishment of T–cell tolerance (30).

Although TIM–3 has been considered to be expressed only by T cells, it is now clear that TIM–3 is expressed by multiple cell types, including myeloid cells (31). In our results, the surface expression of TIM–3 on neutrophils was not detected using the flow cytometry technique, however intracellular TIM–3 expression was detectable in neutrophils isolated from patients with RA. Even if the precise role of TIM–3 in the neutrophils isolated from RA patients was not defined in the present study, it is possible that CEACAM1 and TIM–3 can be co–ordinately regulated in patients with RA. Many pathogens utilize CEACAM1 as a receptor on neutrophils resulting in a potential inhibition of the inflammatory responses. In fact, a feedback loop provided by CEACAM1 was demonstrated to downregulate inflammasome activation in neutrophils (32). However, CEACAM1–TIM–3 interaction in neutrophils could be highly complex. Further and careful investigations are required to delineate the underlying molecular mechanisms concerning the changes in CEACAM1 expression and its intracellular signaling in RA.

There are several limitations in this study. The sample size was relatively small and the numbers of RA patients with disease activity were limited. A second limitation is the restricted number of cell types were analyzed by flow cytometry with a major focus on the immune compartment, so that additional studies would be needed to analyze the more detailed lymphoid and myeloid lineage cells. The protein interactions between CEACAM1 and TIM–3 were not investigated in this study.

In summary, our data indicate that CEACAM1 is expressed in peripheral blood neutrophils in patients with RA and that its expression was upregulated in response to rheumatoid inflammation. Inflammation-related cell-surface expression of CEACAM1 on neutrophils, in the context of a rheumatoid environment, might be implicated with the rheumatoid inflammatory process.

## Data availability statement

The raw data supporting the conclusions of this article will be made available by the authors, without undue reservation.

## Ethics statement

The studies involving human participants were reviewed and approved by ethics committee of Fukushima Medical University. Written informed consent to participate in this study was provided by the participants’ legal guardian/next of kin.

## Author contributions

HM, YF, KS, YS, SY, NM, JT, MY-F, TA, SS, ES, and HW were involved in the acquisition of the clinical data. HM, YF, and KM drafted the manuscript and carried out the molecular biochemical studies. MO and TM contributed as an advisor to study protocols of flow cytometry. HM, YF, MO, and KM participated in the design of the study. HM and YF performed the statistical analysis. All authors contributed to the article and approved the submitted version.
