# Supplementary figures and images for: Increased CEACAM1 expression on peripheral blood neutrophils in patients with rheumatoid arthritis
Source: Front Immunol. 2022 Dec 14;13:978435. doi: 10.3389/fimmu.2022.978435 (PMC9794574; doi:10.3389/fimmu.2022.978435)

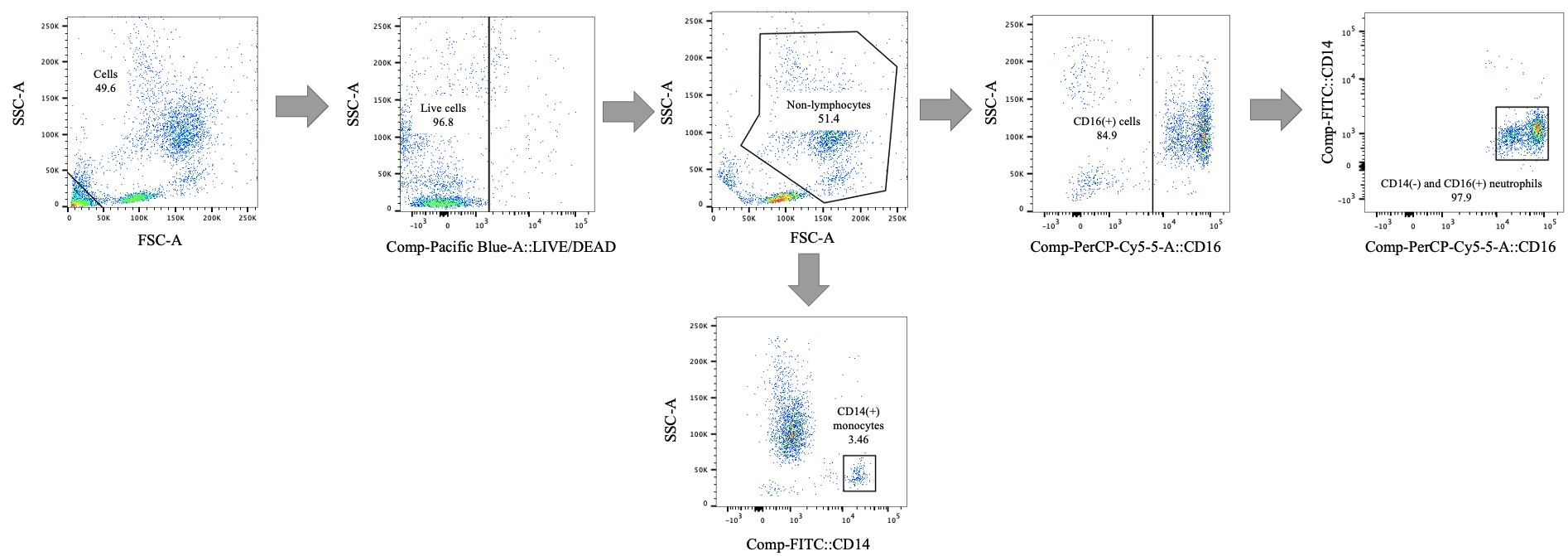

Supplement: Supplementary file 1 [file Image_1.tiff]

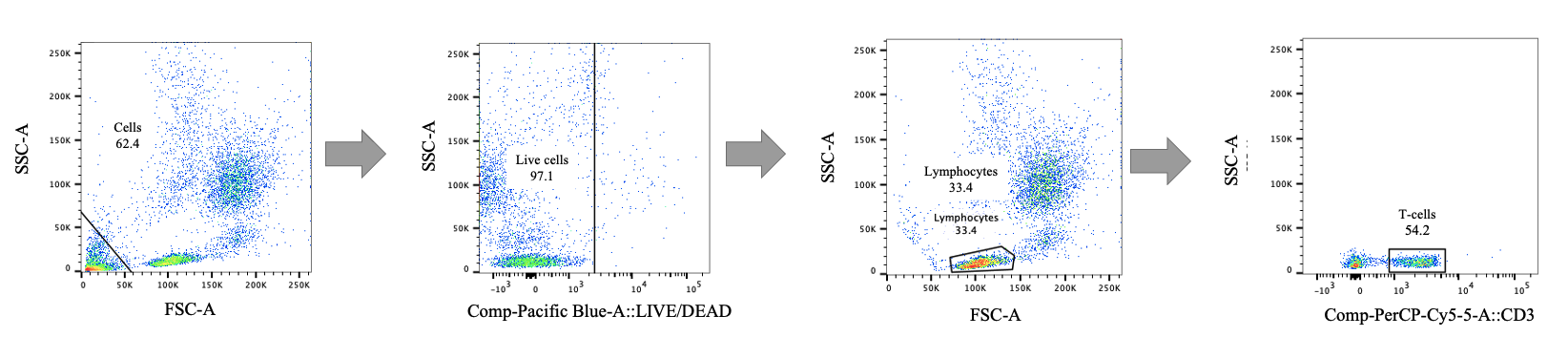

Supplement: Supplementary file 2 [file Image_2.tiff]

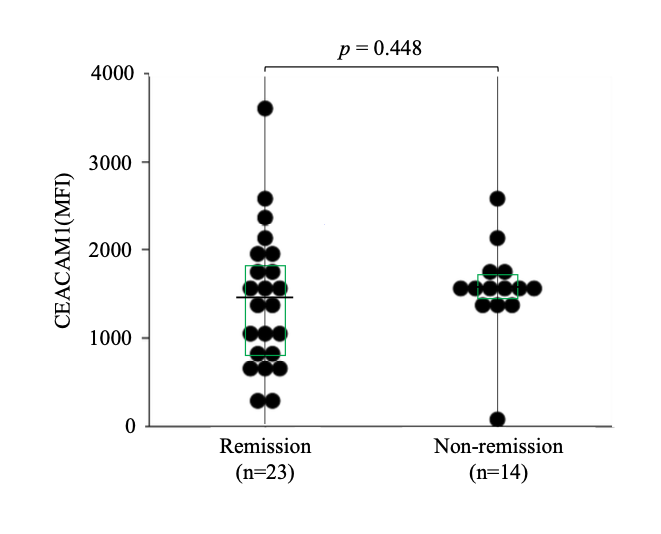

Supplement: Supplementary file 3 [file Image_3.tiff]

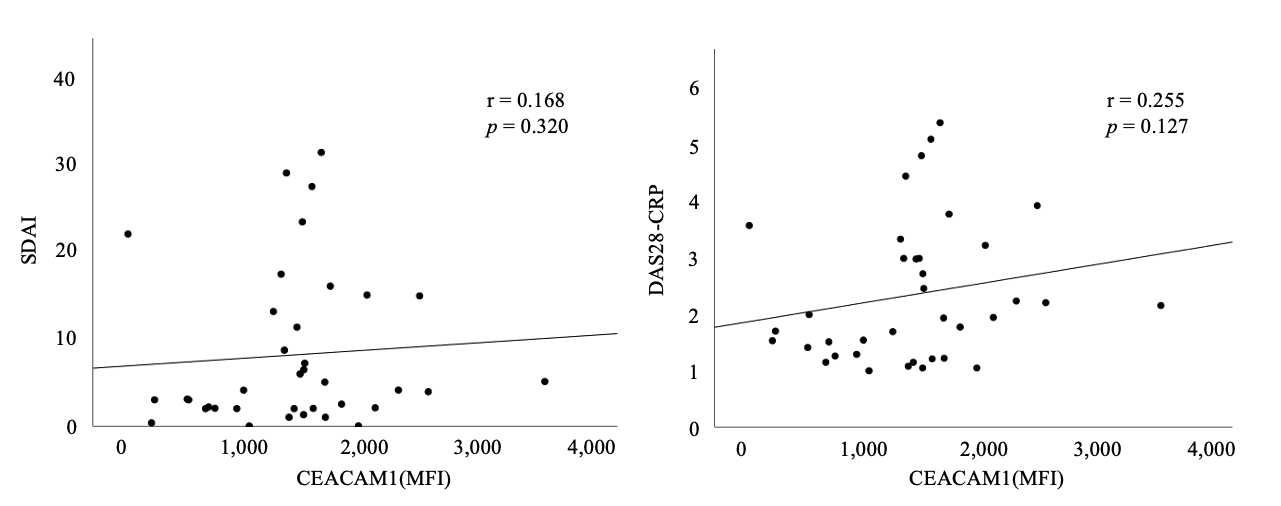

Supplement: Supplementary file 4 [file Image_4.tiff]

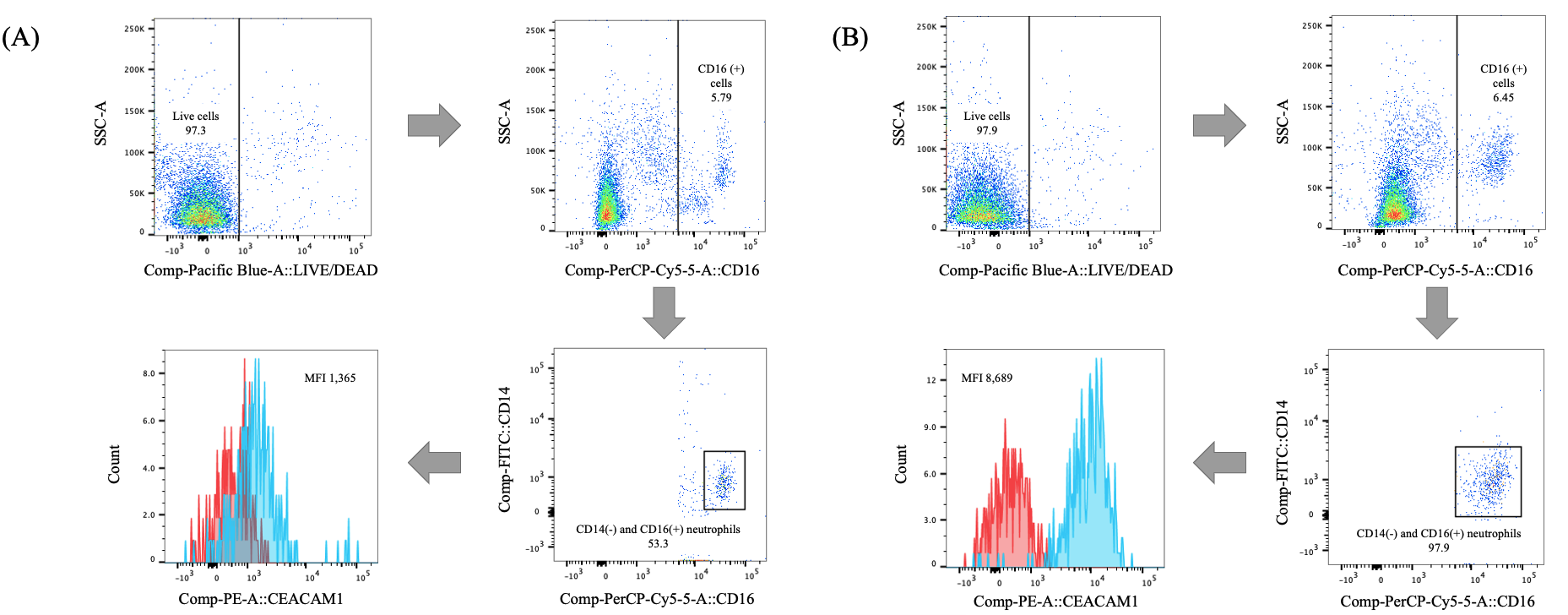

Supplement: Supplementary file 5 [file Image_5.tiff]
